# Value of high-speed videoendoscopy as an auxiliary tool in differentiation of benign and malignant unilateral vocal lesions

**DOI:** 10.1007/s00432-023-05543-y

**Published:** 2024-01-13

**Authors:** Jakub Malinowski, Wioletta Pietruszewska, Magdalena Kowalczyk, Ewa Niebudek-Bogusz

**Affiliations:** Department of Otolaryngology, Head and Neck Oncology, Medical University of Lodz, Lodz, Poland

**Keywords:** Organic dysphonia, Glottic cancer, High-speed videoendoscopy, Kymography, Objectivization, Objective parameters

## Abstract

**Purpose:**

The study aimed to assess the relevance of objective vibratory parameters derived from high-speed videolaryngoscopy (HSV) as a supporting tool, to assist clinicians in establishing the initial diagnosis of benign and malignant glottal organic lesions.

**Methods:**

The HSV examinations were conducted in 175 subjects: 50 normophonic, 85 subjects with benign vocal fold lesions, and 40 with early glottic cancer; organic lesions were confirmed by histopathologic examination. The parameters, derived from HSV kymography: amplitude, symmetry, and glottal dynamic characteristics, were compared statistically between the groups with the following ROC analysis.

**Results:**

Among 14 calculated parameters, 10 differed significantly between the groups. Four of them, the average resultant amplitude of the involved vocal fold (AmpInvolvedAvg), average amplitude asymmetry for the whole glottis and its middle third part (AmplAsymAvg; AmplAsymAvg_2/3), and absolute average phase difference (AbsPhaseDiffAvg), showed significant differences between benign and malignant lesions. Amplitude values were decreasing, while asymmetry and phase difference values were increasing with the risk of malignancy. In ROC analysis, the highest AUC was observed for AmpAsymAvg (0.719; *p* < 0.0001), and next in order was AmpInvolvedAvg (0.70; *p* = 0.0002).

**Conclusion:**

The golden standard in the diagnosis of organic lesions of glottis remains clinical examination with videolaryngoscopy, confirmed by histopathological examination. Our results showed that measurements of amplitude, asymmetry, and phase of vibrations in malignant vocal fold masses deteriorate significantly in comparison to benign vocal lesions. High-speed videolaryngoscopy could aid their preliminary differentiation noninvasively before histopathological examination; however, further research on larger groups is needed.

**Supplementary Information:**

The online version contains supplementary material available at 10.1007/s00432-023-05543-y.

## Introduction

One of the first symptoms presented by patients with glottic organic lesions is dysphonia. Such complaints require a diagnostic process that includes visualization of the larynx, including assessment of the lesion’s morphology, by means of videoendoscopy (Dejonckere et al. 2001; Hald et al. 2023). An adjunct to this method, enabling functional assessment, are advanced diagnostic techniques, utilizing improved image capture and lighting such as laryngovideostroboscopy (LVS), narrow band imaging (NBI), and recently high-speed videoendoscopy (HSV) (Schade and Müller 2005; Svec et al. 2007; Woo 2014). Those techniques enable precise visualization of laryngeal structure and function allowing the diagnosis of both functional and organic dysphonia (Bohr et al. 2013, 2014).

However after detection of organic lesions of the glottis, the most important is the differential diagnosis between benign and malignant lesions (Sigston et al. 2006; Gandhi et al. 2021). The golden standard remains clinical assessment with the establishment of the initial diagnosis, followed by a surgical approach—either biopsy or complete removal of the lesion with subsequent determination of the final diagnosis based on postoperative histopathology. However, there exist several tools able to assist the physician in the decision-making process—especially in the initial assessment and deciding to perform either a biopsy or total surgical removal of the lesion. Those devices (among others) are NBI, LVS, and HSV. Researchers leading their constant development are attempting to use HSV in the visualization of vocal fold oscillations in organic lesions (Yamauchi et al. 2021; Kaluza et al. 2022).

In NBI technique, the light source emits narrow bands of light, corresponding to the absorption of hemoglobin, providing enhanced imaging of mucosal capillaries, allowing more precise assessment of its structure. As cancerous growth promotes blood vessels proliferation, there exist characteristic patterns, observed in malignant lesions, classified by (Ni et al. 2011; Sun et al. 2017).

The use of LVS for organic lesions has been established by previous research in this area (El-Demerdash et al. 2015; Rzepakowska et al. 2017; Itigi et al. 2022). Unfortunately, this technique is susceptible to failure in some of the vocal fold organic masses, as it requires synchronization of strobe light with the fundamental frequency of the patient’s voice. Achieving this synchronization is difficult in short and asynchronous phonations, commonly found in patients with organic lesions. HSV, on the other hand, was until recently used mainly for research purposes and for diagnosing functional disorders of the larynx (assessment of kymographic sections was mostly qualitative, subjective analysis by experienced clinicians) (Tsutsumi et al. 2017; Woo 2020; Kosztyła-Hojna et al. 2021; Schlegel et al. 2021). Development of the HSV technique allowed to overcome those difficulties: eliminated most technical problems with the recording process and provided advanced methods of data analysis—including objective, quantitative assessment of vocal fold movement (Woo 2014; Zacharias et al. 2018; Kist et al. 2021a; Malinowski et al. 2021). Thus, more researchers tend to utilize HSV kymographic analysis in organic lesions of the glottis, yielding promising results (Powell et al. 2016, 2020; Gandhi et al. 2021). Despite the evolution of HSV, not all of the existing problems have been solved, especially involving the choice of parameters evaluating phonatory oscillations (Fehling et al. 2020; Mohd Khairuddin et al. 2021).

Quantitative analysis of kymographic cross-sections allows the calculation of four main groups of parameters assessing vocal fold oscillations—amplitude measures, glottal dynamic characteristics, symmetry measures, and perturbation measures (Sielska-Badurek et al. 2019; Krasnodębska et al. 2019; Schlegel et al. 2021). The first three groups describe features of vocal fold movement in the geometrical area of the glottis (spatial analysis), mainly: amplitude, open quotient, asymmetry, and phase difference. Obtained values describe properties common for all vocal cycles in analyzed phonation, focusing on the course of individual vocal cycles. As parameters included among those groups describe similar features of glottal oscillations, we decided to further describe those three groups together as so-called short-term variability (STV) parameters. STV parameters describe features common for all analyzed glottal cycles, focusing on individual cycle. Those values are not exclusive to HSV analysis, they could be previously obtained also from kymographic cross-sections derived from LVS. The fourth group—perturbation measures—are parameters describing the regularity of vocal fold oscillation (both frequency and amplitude) in time, focusing more on the temporal aspect of glottal vibrations. In comparison to STV parameters, perturbation measures (so-called long-term variability parameters) describe differences between consecutive glottal cycles. For this reason, their assessment requires analyzing more frames than in STV parameters. Those perturbation measurements could be previously obtained by means of acoustic voice analysis and recently also by assessment of HSV recordings. Values of perturbation measures are used to analyze differences between consecutive vocal cycles, describing the stability of phonation (Schlegel et al. 2019; Malinowski et al. 2023). LVS recordings contain far too few vocal cycles to assess this group of parameters. Furthermore, instead of recording the full glottal cycle, the LVS technique samples points along different vocal oscillations, to recreate its image.

A multitude of parameters and methods of calculation causes problems in selecting the right set of them to apply to clinical purposes. Thus, currently most research effort concentrates on developing a standardized HSV investigation protocol and data analysis tools, facilitating preliminary, non-invasive detection of malignancy among organic lesions of the glottis (Turkmen and Karsligil 2019; Kist et al. 2021b).

The aim of the study was to assess the relevance of objective vibratory parameters derived from HSV kymography (quantified amplitude, symmetry and glottal dynamic characteristics) as a support to videoendoscopy, which assists clinicians in establishing an initial diagnosis of benign and malignant glottal organic lesions before histopathologic examination.

## Materials and methods

### Participants

The study included 175 patients hospitalized in the Department of Otolaryngology, Head and Neck Oncology of the Medical University of Lodz from 09.2020 to 12.2022. The control group consisted of 50 patients: 33 women and 17 men. The inclusion criteria for this group were: > 18 years of age, no dysphonia symptoms at the time of the examination, no history of dysphonia, and no structural or functional abnormalities found in endoscopic examination. Subjects in this group were between 19 and 83 years old, with an average age of 41.52 ± 17.44 years.

The study group consisted of 125 patients. Inclusion criteria for the study group were: > 18 years of age; current dysphonia, presence of unilateral organic lesion in the glottis. Subjects with dysphonia were later divided into two groups. The benign lesions group included 85 participants diagnosed with benign vocal lesions (including polyps, cysts, nodules, and unilateral Reinke’s edema). This group consisted of 58 women and 27 men, aged from 22 to 87 years, with the mean age of 53.28 ± 13.3 years. The second group included 40 patients (30 men and 10 women) diagnosed with early glottic cancer. Participants in this group were aged from 42 to 85 years, with an average age of 67.45 ± 7.01 years. All patients with organic lesions underwent transoral laser microsurgery (TOLMS), resulting in histopathologic confirmation of the initial diagnosis. All the malignant lesions were squamous cell carcinoma G1 or G2, classified clinically as T1 involving one vocal fold.

The exclusion criteria for both groups were inability to obtain recordings with sufficient visualization quality for analysis, and organic lesions involving both sides of the larynx.

Approval for this study was granted by the Ethical Committee of the Medical University of Lodz (no. RNN/96/20/KE 08/04/2020), and all patients gave written informed consent to participate in the research.

### Examination

First, the patients were assessed by an ENT physician who made the initial diagnosis. After that, every patient was subjected to examination by means of a rigid endoscope paired with HSV camera and laser illumination. The examination equipment was supported by a computer with dedicated software allowing for the recording of video signal and storing it for further analysis. Initially, the camera was set at a recording speed of 24 frames per second (fps) to center the video sensor on the glottis and perform a primary assessment of the larynx. Next, the camera was set into high-speed recording mode, and multiple HSV sequences were captured. For this study recording speed was 3200 fps. Each sequence contained 2000 fps, with the full-time length of a single sequence of 625 ms (Malinowski et al. 2021). More details on the technical aspects of HSV recording can be found in our previous publication. Later, quality of the recordings was assessed, and the best and most representative films were selected for each patient for further assessment.

### Analysis

**Fig. 1:**
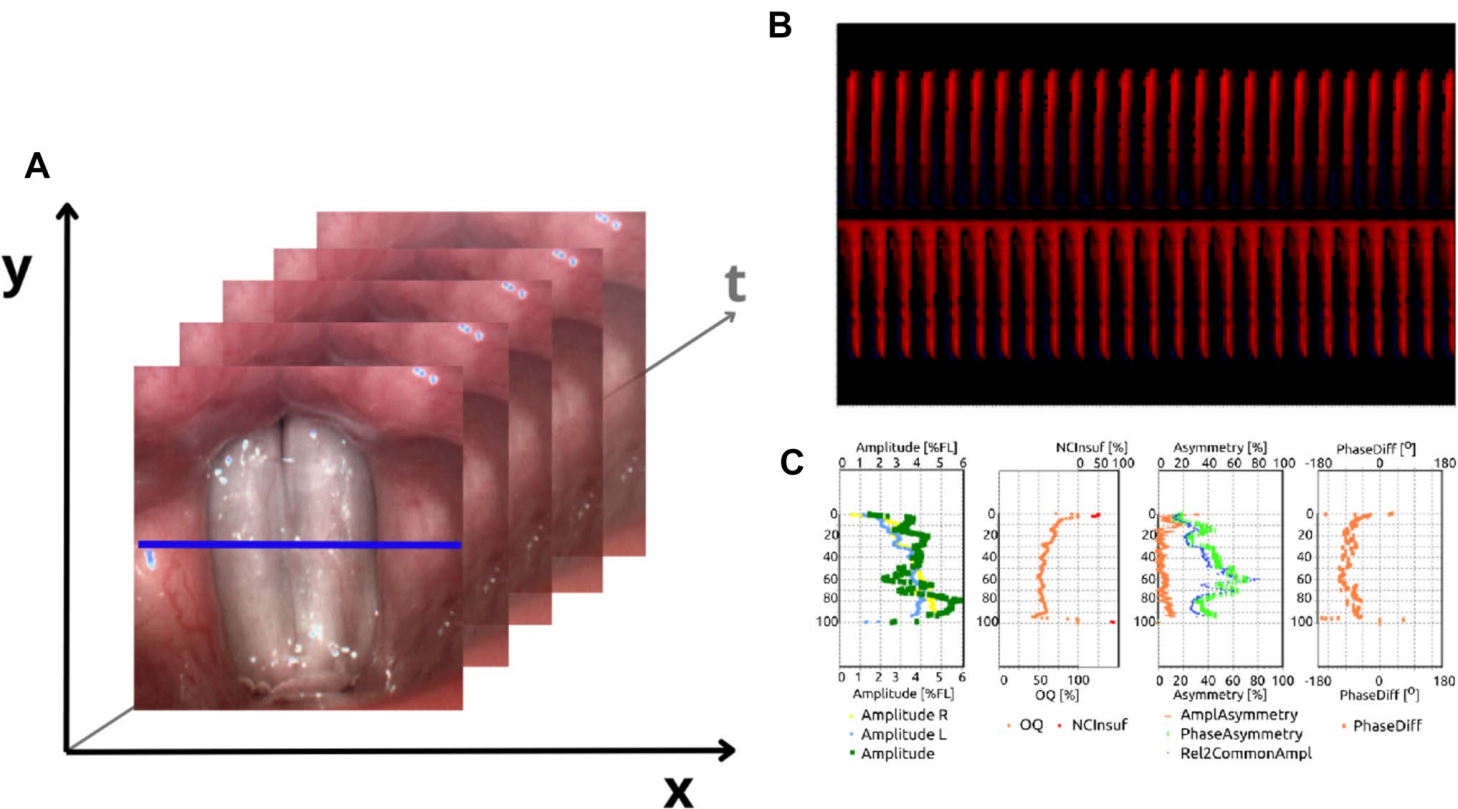
Aspects of HSV analysis: **A** Three axes describing properties of vocal oscillations that can be assessed using different types of HSV analysis. x–mediolateral, y–longitudinal, and t–temporal data. STV analysis focuses on the evaluation of the x- and y-axis—describing geometric aspects of vocal folds oscillation both along and perpendicular to the glottal center in a few cycles, both comparing left and right vocal folds and analyzing resultant features of their vibration. Temporal (perturbation) analysis focuses more on the stability of phonation, analyzing the regularity of both amplitude and frequency in longer periods (y-axis). **B** Phonovibrogram—a diagram presenting the movement of the vocal folds in time in relation to the center of the glottis—the center of the diagram indicates the anterior part of the glottis; the upper part describes the movement of the left vocal fold while lower part describes the movement of the right vocal fold. **C** Values of STV analysis presented in the form of diagrams. From left to right: amplitude measures, glottal dynamic characteristics (open quotient), and two diagrams presenting symmetry measures (first: amplitude and phase asymmetry; second: phase difference)

Detailed kymographic analysis was performed to quantitatively assess vocal fold vibrations. As described by Yamauchi et al. sets of data from HSV kymography can be classified as three dimensions of HSV—mediolateral (x), longitudinal (y), and temporal (t), as shown in Fig. 1 (Yamauchi et al. 2021). The first two—the x- and y-axis—describe spatial properties of glottal oscillations and can be assessed subjectively (visual-perceptual rating), by laryngotopography and by means of HSV (Sakakibara et al. 2010; Tsuji et al. 2014; Yamauchi et al. 2015). The temporal aspect can be evaluated either on its own (by analysis of glottal area and width waveform) or along with mediolateral dimension—using digital kymography (Schlegel et al. 2020; Kist et al. 2021a; Yousef et al. 2023). In this study, to compare involved and healthy vocal folds, we focused on the spatial aspect of HSV analysis (x- and y-axis), resulting in the calculation of STV parameters including amplitude measures, glottal dynamic characteristics, and symmetry measures. Those parameters describe the movement of vocal folds along and perpendicularly to the glottal axis.

First, the software automatically selected a part of the recording for further analysis. Contrary to LVS, in the case of HSV recordings, manual stabilization and centering of the image for each frame was not required, because, in such a short recording time (625 ms), the movement of the endoscope tip is negligible. Later the vocal folds edges were identified semi-automatically—the examiner marked them in five points on a single representative frame, and based on those points, the software indicated them on all frames and along the whole length of the glottis. The examiner was then able to verify and adjust the automated detection in case of any discrepancy. In the final step, the software calculated a set of STV parameters describing characteristics of the movement of vocal folds during the glottal cycle, specifically: amplitude measures, glottal dynamic characteristics, and symmetry measures. Amplitude measures include four parameters: average amplitude (AmpAvg), the average amplitude of the middle third of the glottis (AmpAvg_2/3), the average amplitude of the involved vocal fold (AmpInvolvedAvg), and the average amplitude of the healthy vocal fold (AmpHealthyAvg)—their values indicate the average resultant amplitude of vocal fold movement for the whole glottal gap, its middle third part, involved, and healthy vocal fold, respectively. This parameter is calculated as an averaged value for all points along the length of respective vocal fold. Whenever a parameter below has a suffix “_2/3”, it relates to the middle third part of the glottal length. We decided to include those parameters as the middle part of the glottis which is known to be the location of the maximum amplitude of oscillations, and disruptions in this area tend to have a larger impact on phonation. In our study, organic lesions were always unilateral, so based on the location of the lesion, we compared both folds in a series of analyses. For this purpose, we used AmpInvolvedAvg and AmpHealthyAvg to compare vibrations of healthy and involved vocal folds. An exception was made in the statistical analysis with regard to these parameters—comparison was performed only in benign and malignant lesion groups. Among glottal dynamic characteristics, four parameters are calculated: average open quotient (OQAvg), OQAvg_2/3, relative glottal gap area (RGGA), and non-opening. OQAvg and OQAvg_2/3 describe the average open quotient for the whole glottal gap and its middle third part, respectively, indicating the ratio of the glottal opening phase to the whole length of the vocal cycle. RGGA defines the ratio of minimal to maximal area of the glottis during the cycle, while non-opening indicates part of the glottis without opening during the cycle. Symmetry measures include five parameters: average amplitude asymmetry (AmplAsymAvg), AmplAsymAvg_2/3, average phase asymmetry (PhaseAsymAvg), PhaseAsymAvg_2/3, and absolute average phase difference (AbsPhaseDiffAvg). AmplAsymAvg and AmplAsymAvg_2/3 are coefficients comparing individual amplitudes of both vocal folds movement in relation to each other for the whole glottal gap and its middle third part, respectively. PhaseAsymAvg and PhaseAsymAvg_2/3 are coefficients comparing the sum of individual amplitudes of vocal fold motion to the amplitude of their resultant movement. Finally, AbsPhaseDiffAvg describes the absolute mean value of phase difference for whole vocal folds. The parameters are precisely described in Supplementary Table 1 and were calculated as described by Sielska-Badurek et al. and Just—inventor of the software (Just et al. 2016; Sielska-Badurek et al. 2019).

### Statistical analysis

First, we used the Shapiro–Wilk test to check assumptions regarding the distribution of analyzed variables. Depending on fulfilled assumptions, for two groups of patients (benign–malignant and involved–healthy), either non-parametric *U* Mann–Whitney test or parametric *T* test was performed.

For three groups (norm–benign–malignant), we used a one-way ANOVA test. Depending on fulfilled suppositions, we chose either parametric test to analyze variance or non-parametric Kruskal–Wallis test. If the results of the test showed significant differences between mean values or distribution functions, we performed appropriate post hoc tests: non-parametric multiple comparison test or least significant differences (LSD) test.

Next, we performed a ROC analysis in two variants to check which of the parameters would be a good classifier. To do that, patients were split into two, creating a two-state variable. For the first variant, patients were split into norm and benign + malignant clusters, and for the second variant into benign and malignant clusters. The ROC curve was plotted for each of the parameters and the AUC area was calculated. Results showed which parameters are significant as classifiers. Next, we used Youden index, to determine a cut-off point for each of the parameters. Youden Index is a value giving information on the maximum distance of the points on the ROC curve from the diagonal of the square with sides equal to 1 (red line on the ROC diagrams). For this point, we presented the values of sensitivity and specificity. The parameters’ values were later correlated with the presence and type of lesion, dividing them into either boosters or inhibitors. Values of boosters were increasing along with the risk of disease (either any lesion or malignancy, depending on the variant of ROC analysis as described above), and values of inhibitors were decreasing conversely to the growing risk of disease.

Calculations were performed using STATISTICA.PL software, version 13.3 (Statsoft, Cracov) and Microsoft Excel.

## Results

### Qualitative assessment

**Fig. 2:**
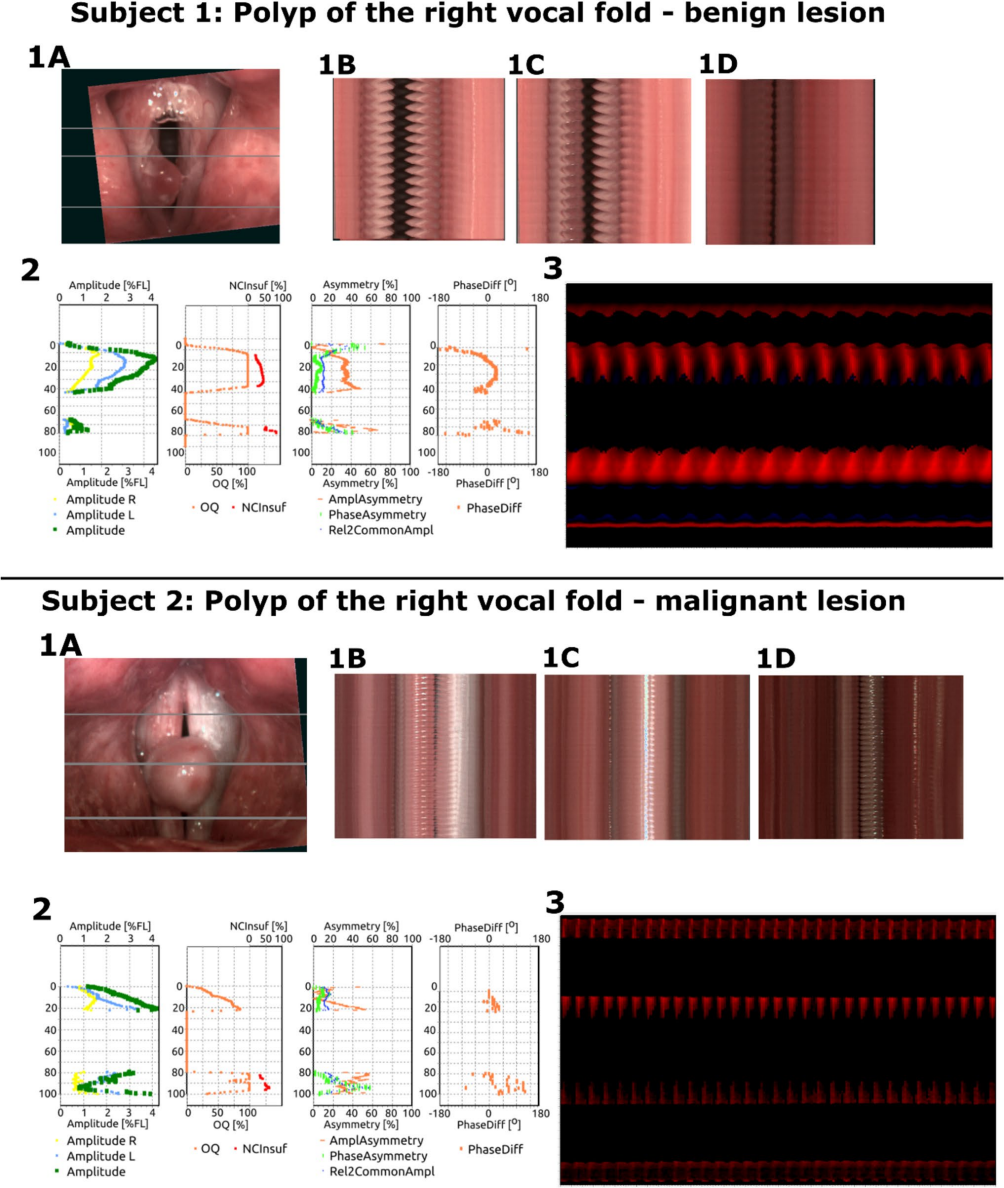
Presentation of HSV STV analysis for the two subjects described in the text. For each subject, the analysis includes **1** the image of the glottis (**1A**) with grey lines presenting three representative kymographic cross-sections for three functional parts of the glottis (from left to right) consecutively posterior (**1B**), middle (**1C**) and anterior (**1D**) part. **2** Values of STV analysis presented in the form of diagrams, along the glottal axis (y-axis on each diagram), from left to right: amplitude measures, glottal dynamic characteristics (open quotient), and two diagrams presenting symmetry measures (first: amplitude and phase asymmetry; second: phase difference). **3** Phonovibrogram—a diagram presenting the movement of the vocal folds in time in relation to the center of the glotti—the center of the diagram indicates the anterior part of the glottis; upper part describes the movement of the left vocal fold while lower part describes the movement of right vocal fold—darker color indicates decreased amplitude (as described in the text)

Aside from numerical values of parameters, also four graphs were generated—one for each parameter group and a phonovibrogram visualizing the movement of vocal fold edges in relation to the glottal axis. In Fig. 2, we present a comparison of analysis results for two patients: Subject 1—a 56-year-old male, complaining of mild to severe dysphonia. Initial examination revealed a polypoid mass on the right vocal fold. Subjective kymographic assessment showed a moderate decrease in amplitude of the involved vocal fold, without significant asymmetry or phase difference. As shown on the phonovibrogram, there was a non-opening area of the glottis encompassing the lesion. The average amplitude of the involved vocal fold reached 2.7%FL and the resultant amplitude was 6.5%FL. Values of average amplitude are expressed in the percentage of the total length of the respective vocal fold (percent of fold length–%FL). Surgical removal of the mass was performed, and the patient achieved satisfying postoperative vocal results. In this case, histopathology confirmed the initial diagnosis. Subject 2—a 69-year-old male, complaining of severe dysphonia, initial examination revealed a large polypoid mass on the right vocal fold. Subjective analysis revealed a significant decrease in the amplitude of the involved vocal fold—especially visible on the phonovibrogram as the dark red color of the lower part of the diagram. On phonovibrograms, brightness increases along with the amplitude of oscillations. There was no significant elevation of asymmetry or phase difference. As noticed in the preoperative HSV examination, in this patient, the average amplitude of the involved vocal fold reached 1.0%FL, and the resultant amplitude was 2.5%FL. Decreased (in comparison to Subject 1) values of amplitude indicated a risk of deep tissue infiltration. A surgical approach (TOLMS) was implemented with the removal of the mass along with its pedicle—postoperative histopathology revealed squamous cell carcinoma with no malignant infiltration of the pedicle and free vocal fold edge. This comparison is an accurate example of how advanced visualization techniques with objective analysis can assist clinicians in establishing initial diagnosis. A detailed description of STV analysis for the two subjects is provided in Fig. 2.

### Objective analysis—parameters

All the parameters were compared in three groups—healthy participants, benign lesion subjects, and malignant lesion subjects. One-way ANOVA test was performed to determine if the distribution of the parameters differs between all three groups altogether. Depending on fulfilled suppositions, we chose either parametric test to analyze variance or non-parametric Kruskal–Wallis test (one-way ANOVA on ranks). Fundamental frequency did not differ between the groups. Among STV parameters, significant diversities between the groups were found for 10 out of 14. Detailed results are shown in Table 1.

**Table 1:** Results of Kruskal–Wallis test for individual vibratory parameters, with exclusion of OQAvg (marked with*)—this measurement’s values had normal distribution and were analyzed with the use of parametric test

Parameter	Diagnosis	*n*	Mean	Median	*p*
AmpAvg	Norm	50	10.43400	8.25000	0.0000
	Benign	85	7.1824	6.2000	
	Malignant	40	5.62250	4.85000	
AmpAvg_2/3	Norm	50	12.18600	10.30000	0.0000
	Benign	85	7.8494	6.7000	
	Malignant	40	6.07750	5.25000	
AmpInvolvedAvg	Benign	85	3.92941	3.40000	0.0003
	Malignant	40	2.62750	2.20000	
AmpHealthyAvg	Benign	85	4.84000	4.20000	0.0775
	Malignant	40	4.32000	3.65000	
Non-closing	Norm	50	16.71400	7.70000	0.9759
	Benign	85	16.9318	7.6000	
	Malignant	40	15.66500	13.75000	
Non-opening	Norm	50	3.39600	0.30000	0.0024
	Benign	85	10.3624	2.9000	
	Malignant	40	12.49500	5.85000	
RGGA	Norm	50	7.72200	3.25000	0.1128
	Benign	85	14.0341	4.1000	
	Malignant	40	15.20500	9.75000	
OQAvg	Norm	50	62.48400	59.95000	0.0793*
	Benign	85	56.0271	57.6000	
	Malignant	40	53.45500	50.85000	
OQAvg_2/3	Norm	50	66.33400	63.50000	0.0092
	Benign	85	54.1047	53.8000	
	Malignant	40	51.98500	47.60000	
AmplAsymAvg	Norm	50	11.48600	10.55000	0.0000
	Benign	85	21.5353	18.6000	
	Malignant	40	32.23500	32.30000	
AmplAsymAvg_2/3	Norm	50	9.60800	8.50000	0.0000
	Benign	85	20.4647	18.0000	
	Malignant	40	31.05250	32.00000	
PhaseAsymAvg	Norm	50	12.21400	6.40000	0.0000
	Benign	85	18.1341	14.0000	
	Malignant	40	18.31000	17.50000	
PhaseAsymAvg_2/3	Norm	50	12.07600	6.05000	0.0033
	Benign	85	18.3306	12.3000	
	Malignant	40	17.83750	14.90000	
AbsPhaseDiffAvg	Norm	50	37.72200	31.90000	0.0000
	Benign	85	54.5729	48.2000	
	Malignant	40	70.67750	72.60000	

The table presents mean and median values in each diagnosis group (norm, benign, and malignant). N means the number of cases in each group

**Fig. 3:**
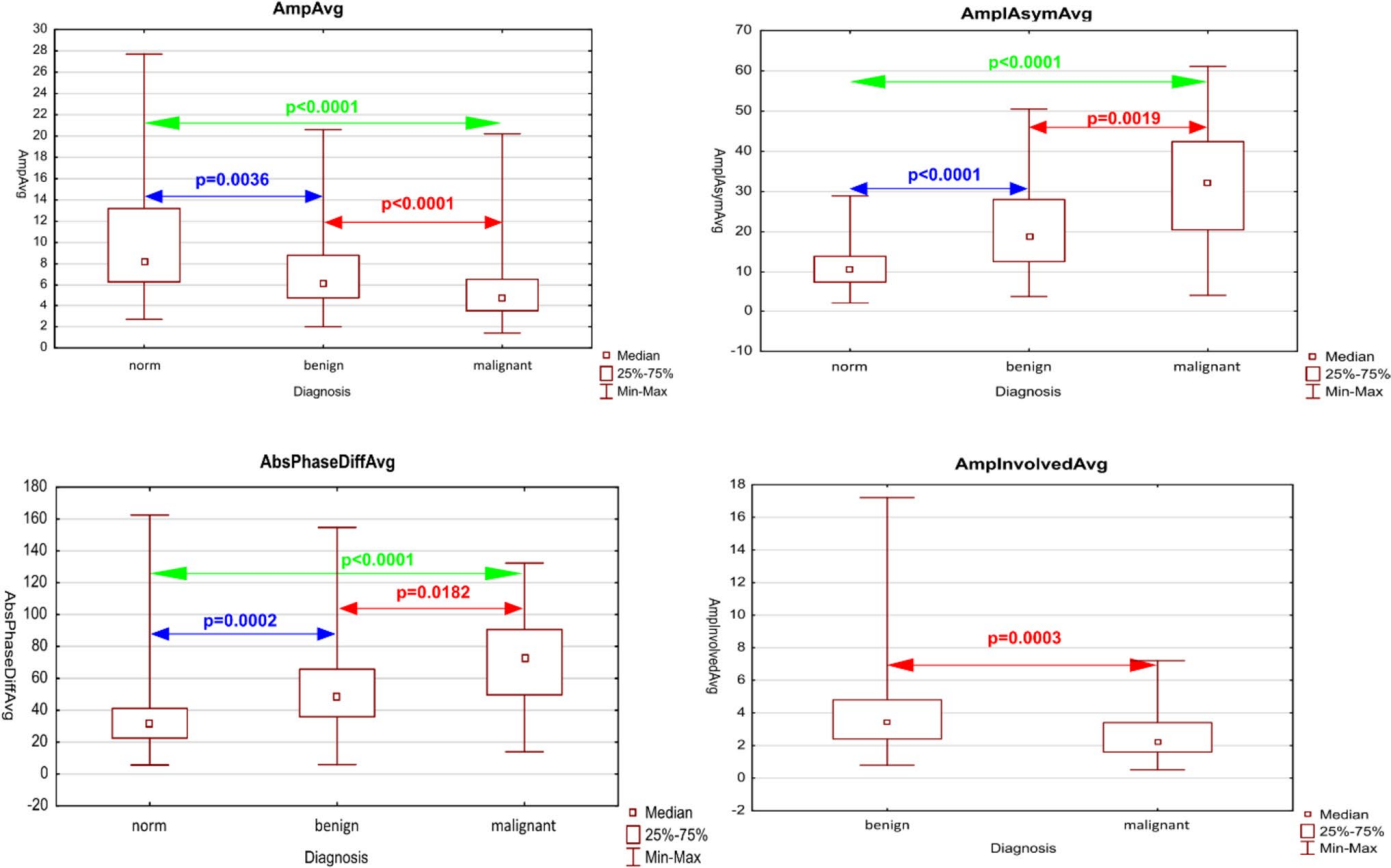
Diagrams presenting median values of individual vibratory parameters in groups divided by diagnosis, specifically: norm, benign, and malignant. Colored arrows present *p* values in bilateral comparisons between the groups: green–norm vs malignant, blue–norm vs benign, and red–benign vs malignant. The parameters presented in this figure differ in a significant way not only between normophonic patients and any organic lesion but also between benign and malignant glottal lesions

Next, we performed a bilateral multiple comparison test to find out if the parameters are able to assess subjects between norm, benign, and malignant lesion groups, giving hope to use them as a predictive parameter. In our previous studies (Malinowski et al. 2023), we found out that there are more parameters able to facilitate the detection of the presence of any organic lesion (both benign and malignant), than to facilitate the prediction of malignancy—demonstrating statistically significant differences between benign and malignant organic lesion. Out of the ten STV parameters demonstrating significant differences between all the groups, we determined that four of them also show differences between benign and malignant lesion groups. Those parameters were: AmpAvg, AmpInvolvedAvg, AmpAsymAvg, and AbsPhaseDiffAvg. The values of amplitude were decreasing from benign to malignant lesions, on the other hand, asymmetry and phase difference were increasing with the appearance of malignancy. Results for those parameters are shown in Fig. 3. The remaining six parameters: (AmpAvg_2/3, non-opening, OQAvg_2/3, AmpAsymAvg_2/3, PhaseAsymAvg, PhaseAsymAvg_2/3) presented similar mathematical trends—decreasing values of amplitude and open quotient and increasing values of asymmetry along with the risk of malignancy, however without reaching statistical significance between benign and malignant lesion groups. Significant differences were found only between healthy subjects and whole organic lesion groups (both benign and malignant altogether).

**Fig. 4:**
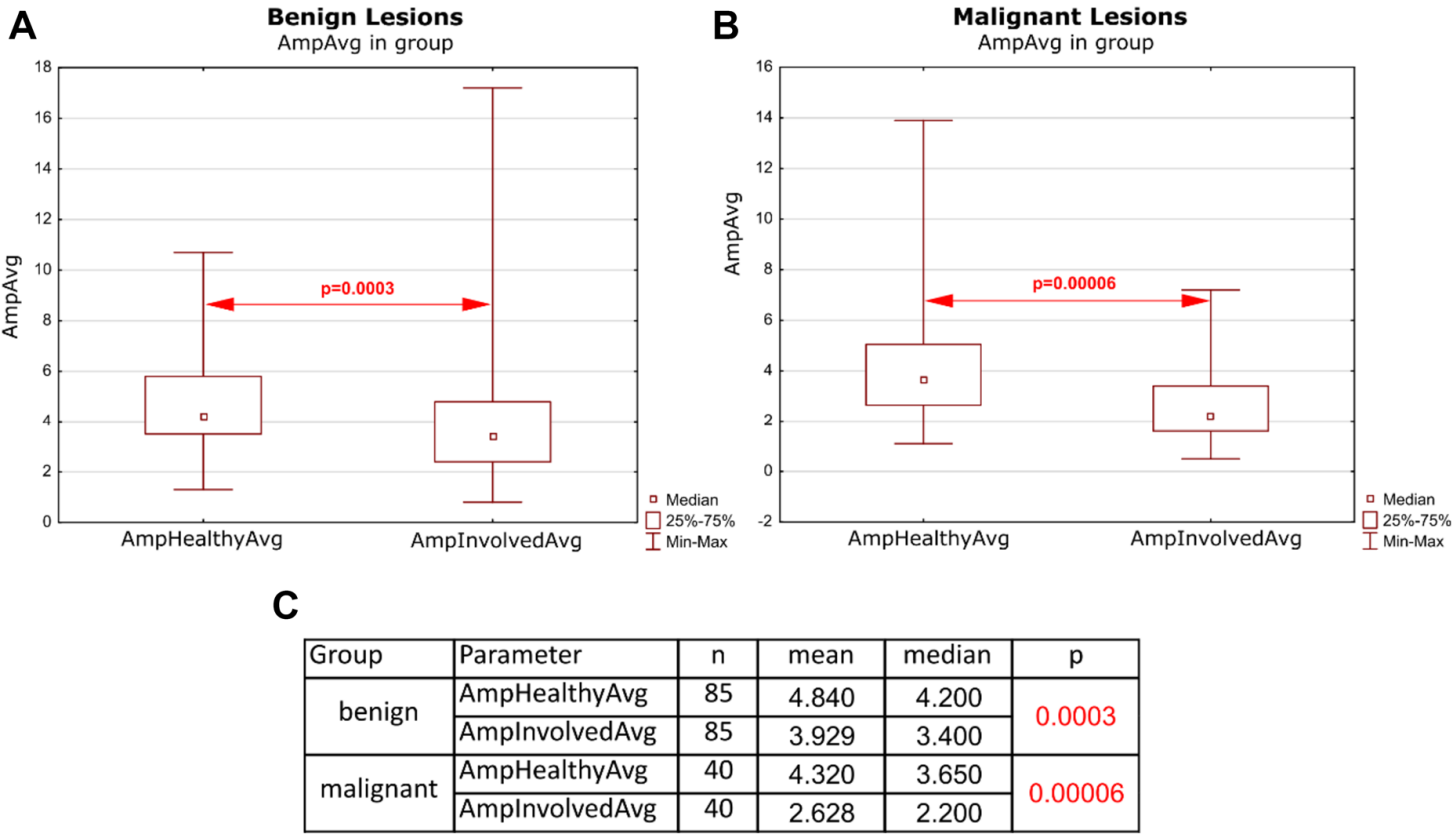
Diagrams presenting median values of average Aaplitude parameter for the healthy and involved fold, respectively. Diagrams are divided by diagnosis, specifically: benign (**A**) and malignant (**B**). Red arrows present the *p* value in comparison between the groups: benign vs malignant. Table **C** presents the results of statistical analysis for those parameters

The parameters comparing involved and healthy vocal fold were subjected to another analysis, comparing values of parameters between involved and healthy vocal folds individually among benign and malignant lesions. We found that the amplitude of healthy vocal folds displayed a higher resultant amplitude than folds involved with organic lesions. This difference was significant for both benign and malignant lesions (Fig. 4).

**Fig. 5:**
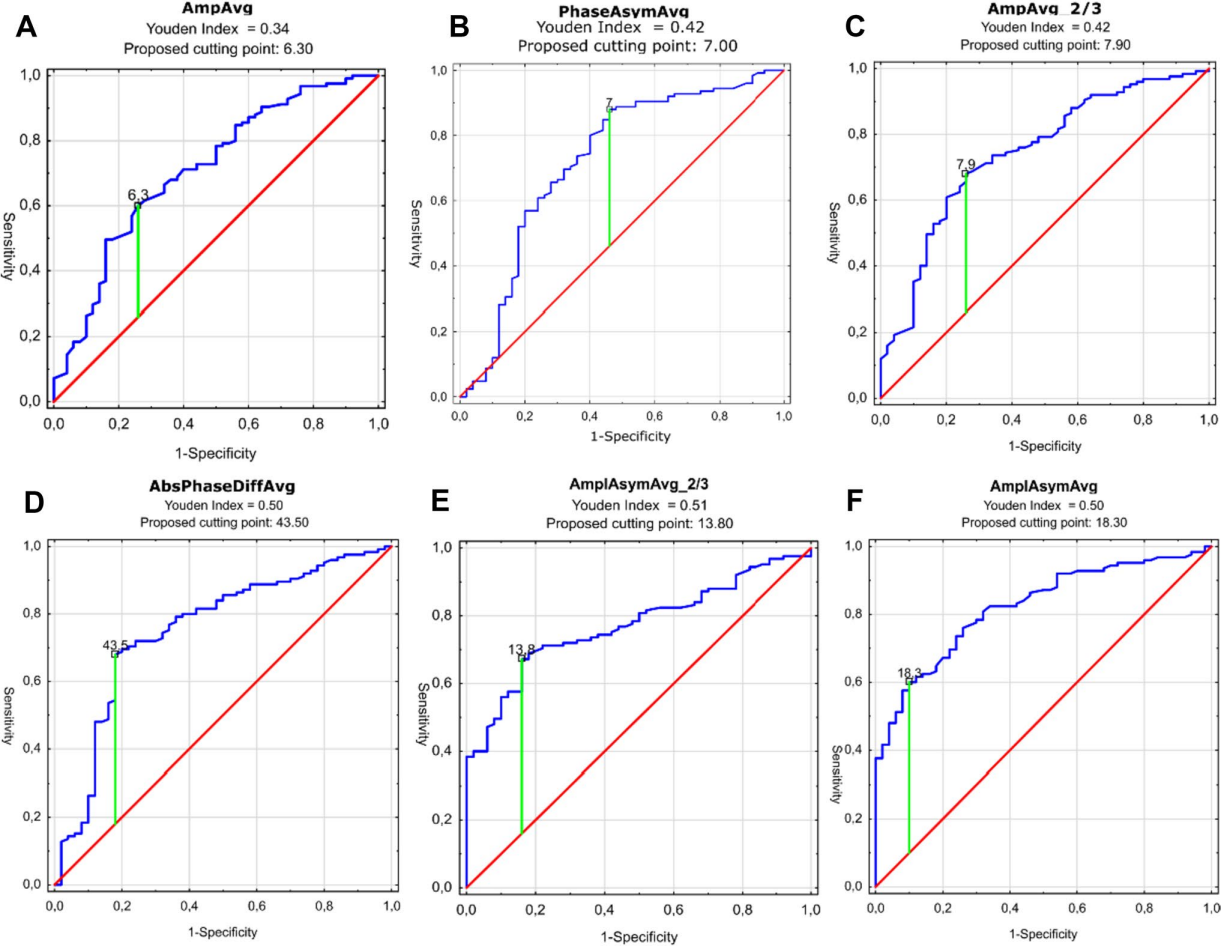
ROC curves for the six best-performing vibratory parameters differentiating normophonic subjects from patients with any organic lesion of the glottis (both benign and malignant) (blue line, **A**–**F**). X- and y-axis present sensitivity and 1-specificity of points on the curve. The red line presents an AUC value of 0.5; the green line presents the value of the Youden index ending in the black square on the curve—the proposed cutting point as described in the text and Table 2

In the next step, we performed AUC ROC analysis in two variants. In the first variant, we assessed the performance of the parameters in detecting the presence of organic lesions. The best performance was observed for AmplAsymAvg, reaching an AUC of 0.822 with 60% sensitivity and 90% specificity (*p* < 0.0001). Altogether six parameters reached AUC values above 0.7, with varying sensitivity and specificity as shown in Table 2. The highest sensitivity was reached by PhaseAsymAvg with an AUC of 0.721, sensitivity of 88%, and specificity of 54%, *p* < 0.0001. On the other hand, the highest specificity was reached by OQAvg_2/3 with an AUC of 0.647, sensitivity of 42%, and specificity of 92%. Detailed results are shown in Table 2 and ROC curves are shown in Fig. 5. The parameters can also be assessed as either boosters or inhibitors. Values of boosters were increasing along with the risk of organic lesion and values of inhibitors were decreasing conversely to the growing risk of lesion. The study showed that inhibitors were amplitude and open-quotient-related parameters, meaning that both the amplitude and opening of vocal folds were decreased in malignant lesions. Boosters were asymmetry and phase difference parameters—those properties of the glottal movement were increased in case of malignancy. For each parameter, by means of the Youden Index, a cutting point was calculated. It was a proposed value for the detection of the presence of any organic lesion. Depending on booster or inhibitor properties of the parameter, its values above (for booster) or below (for inhibitor) the cutting point suggest the presence of an organic lesion; on the other hand, values below (for booster) or above (for inhibitor) indicate normophonic patient, respectively. The sensitivity and specificity of the stated cutting point are also shown in the table.

**Table 2:** Comparison of vibratory parameters (achieving AUC of more than 0.7) in differentiation of normophonic subjects from patients with any organic lesion of the glottis—results of AUC ROC analysis

Parameter	Booster/inhibitor	AUC	AUC lower 95%	AUC upper 95%	*p* value	Youden index	Cutting point	Sensitivity	Specificity
AmplAsymAvg	Booster	0.822	0.76	0.884	0.0000	0.50	18.3	0.6	0.9
AmplAsymAvg_2/3	Booster	0.772	0.704	0.841	0.0000	0.51	13.8	0.67	0.84
AbsPhaseDiffAvg	Booster	0.754	0.671	0.837	0.0000	0.50	43.5	0.68	0.82
AmpAvg_2/3	Inhibitor	0.743	0.662	0.824	0.0000	0.42	7.9	0.68	0.74
PhaseAsymAvg	Booster	0.721	0.629	0.814	0.0000	0.42	7	0.88	0.54
AmpAvg	Inhibitor	0.71	0.624	0.796	0.0000	0.34	6.3	0.6	0.74
OQAvg_2/3	Inhibitor	0.647	0.566	0.727	0.0004	0.34	45.2	0.42	0.92

Exclusion is OQAvg_2/3 (in green), this parameter achieved the highest specificity. Parameters are divided into boosters and inhibitors as explained in the text. Cutting point—calculated with the use of the Youden index, a proposed value for the detection of organic lesions. For boosters values, above the cutting point suggest the presence of organic lesions; on the other hand, values below indicate normophonic patient. The opposite behavior is observed for inhibitors

**Fig. 6:**
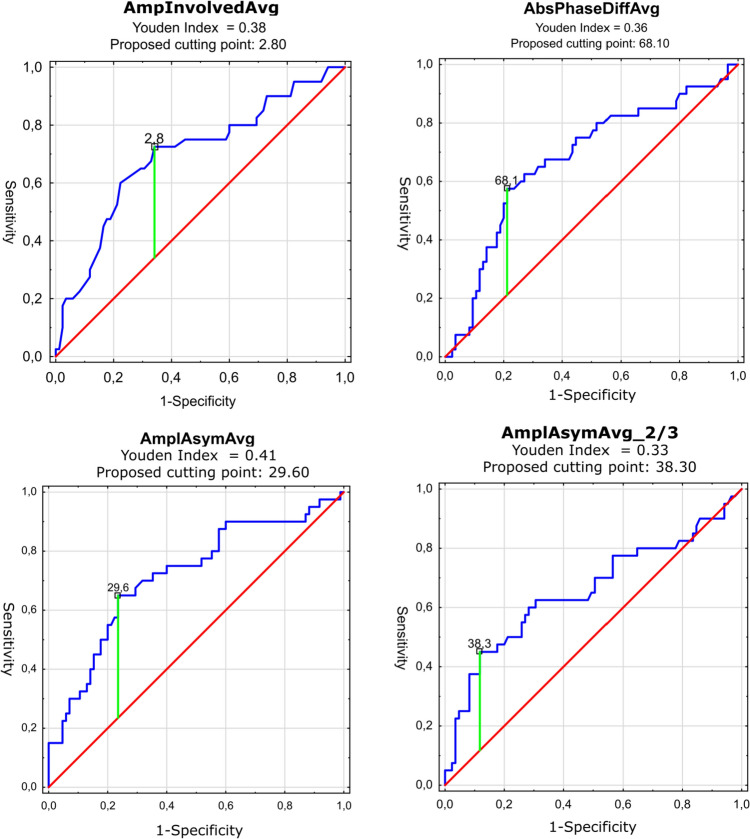
ROC curves for the four vibratory parameters differentiating benign from malignant lesions of the glottis (blue line). X- and y-axis present sensitivity and 1-specificity of points on the curve. The red line presents an AUC value of 0.5; the green line presents the value of the Youden index ending in the black square on the curve—the proposed cutting point as described in the text and Table 3

In the second variant of ROC analysis, we compared the performance of the parameters in the preliminary detection of malignancy among organic lesions. AUC values in this variant were generally lower. The four parameters with the largest values were: AmpInvolvedAvg, AbsPhaseDiffAvg, AmplAsymAvg, and AmplAsymAvg_2/3. The greatest value of AUC was reached by AmplAsymAvg: 0.719 with a sensitivity of 65% and specificity of 76.5% (*p* < 0.0001). The highest sensitivity was achieved by AmpInvolvedAvg with an AUC of 0.7, sensitivity of 72.5%, and specificity of 65.9% (*p* = 0.0002). On the other hand, the highest specificity was reached by AbsPhaseDiffAvg with an AUC of 0.676, sensitivity of 57.5%, and specificity of 78.8% (*p* = 0.0009). Detailed results are shown in Table 3 and Fig. 6. As mentioned above, the parameters displayed properties of either boosters or inhibitors with the same types of parameters belonging to each cluster as in the first variant of ROC analysis. Cutting point was calculated for each parameter as described above.

**Table 3:** Comparison of vibratory parameters in differentiating benign from malignant lesions of the glottis—results of AUC ROC analysis for chosen parameters (achieving AUC of more than 0.65)

Parameter	Booster/inhibitor	AUC	AUC lower 95%	AUC upper 95%	*p*	Youden index	Cutting point	Sensitivity	Specificity
AmplAsymAvg	Booster	0.719	0.619	0.818	0.0000	0.41	29.6	0.650	0.765
AmpInvolvedAvg	Inhibitor	0.70	0.59	0.80	0.0002	0.38	2.8	0.725	0.659
AbsPhaseDiffAvg	Booster	0.676	0.572	0.780	0.0009	0.36	68.1	0.575	0.788
AmplAsymAvg_2/3	Booster	0.655	0.543	0.767	0.0067	0.33	38.3	0.450	0.882

Parameters are divided into boosters and inhibitors as explained in the text. Cutting point–calculated with the use of the Youden index, a proposed value for the detection of organic lesions. For boosters values, above the cutting point suggest the presence of a malignant lesion; on the other hand, values below indicate the presence of a benign lesion. The opposite behavior is observed for inhibitors

## Discussion

High-speed videoendoscopy is an innovative, promising technique for vocal fold vibratory assessment, giving hope to surpass LVS, especially in the assessment of organic lesions, the latter being inapplicable to non-sustained phonations or moderate to severe dysphonia. The evaluation of vocal fold vibrations plays a pivotal role in the detection and diagnosis of glottal organic lesions, being able to assist the clinician in the decision-making process, possibly paving the way for the so-called “optical biopsy” (Volgger et al. 2017; Mehlum et al. 2020). This study aimed to assess the relevance of vibratory parameters (of vocal fold vibrations) quantified amplitude, symmetry, and glottal dynamic characteristics derived from HSV kymography in benign and malignant vocal fold lesions as a preliminary prediction before receiving histopathology results.

We compared parameters evaluating vocal fold vibrations in patients divided into three groups depending on the diagnosis: normophonic group, benign lesion group, and malignant lesion group, with unilateral organic lesions of the glottis. First, we compared the study group of 125 subjects diagnosed with unilateral organic lesions of vocal folds to the control group of 50 normophonic subjects. In the next step, we compared 40 subjects diagnosed with malignant and 85 subjects with benign vocal lesions. Diagnosis of all the organic lesions was confirmed histopathologically. In the study, we aimed to compare vibratory characteristics of healthy and involved vocal fold; therefore, we decided to include only unilateral masses of glottis. We observed in HSV imaging that benign lesions were usually soft, resulting in slight altering of the involved vocal fold’s elasticity and pliability. In cancerous lesions, on the other hand, the infiltration leads to an increase in stiffness of the vocal fold, leading to a greater decrease in amplitude of oscillations (or even absence of phonatory movement) in comparison with benign masses. This decrease in amplitude was shown on the phonovibrogram as the dark red color of the lower part of the diagram—describing the involved vocal fold.

The kymographic analysis of HSV recordings enables an objective assessment of vocal fold oscillations. Sets of data from HSV kymography, as mentioned above and described by Yamauchi et al. can be classified as three dimensions of HSV—mediolateral (x), longitudinal (y), and temporal (t) (Yamauchi et al. 2021). In our previous work, we focused mostly on perturbation measures; however, no parameter was able to singly differentiate benign and malignant glottal lesions—only multivariable analysis allowed selection of statically significant parameters, yielding promising results (Malinowski et al. 2023). In this study, we decided to focus on the STV parameters (x and y dimensions) and evaluate their proficiency in the prediction of both benign and malignant organic glottal lesions.

Our study determined that the parameters able to differentiate normophonic and dysphonic patients (detecting presence of organic glottal lesions) in our study were: AmpAvg, AmpInvAvg, AmpAvg_2/3, Non-opening, OQAvg_2/3, AmpAsymAvg_2/3, AmpAsymAvg, PhaseAsymAvg, PhaseAsymAvg_2/3, AbsPhaseDiffAvg. The presence of an organic lesion causes disruptions of vocal oscillation, reflected in the parameter values. Specifically, the presence of a lesion caused a decrease in average amplitude and open quotient. On the other hand, asymmetry, phase difference, and non-opening parameters were increased. Involved vocal fold due to the effect of the lesion’s mass and infiltration tends to become less elastic and its vibrations are decreased—the effect increases from soft benign lesions to stiff and infiltrative cancerous masses. It can be especially noticed in values of AmpInvolvedAvg—parameter describing specifically the amplitude of vocal fold involved with the lesion, taking into account asymmetry of the material property between involved and non-involved vocal fold (tissue stiffness) (Colden et al. 2001; Gugatschka et al. 2008).

Among glottal dynamic characteristics. Open quotient for the middle part of the glottis and non-opening percentage showed significant differences between normophonic subjects and patients with any organic lesion, while no significant difference was found between malignant and benign masses. A possible interpretation is that the glottal insufficiency caused by the presence of any hypertrophic pathology is similar between both groups and may have resulted from entrapment of a polypoid mass between the free edges of the vocal fold (which could also explain the non-opening percentage). The reason the open quotient was significant only for the middle part of the glottis can be explained by the location of lesions in studied patients: most of the lesions were located near the middle of vocal fold length.

In the available literature, there are limited studies making an attempt at differentiation of benign and malignant lesions based on HSV-derived parameters. However, (Patel et al. 2008) reported rates of successful vibratory assessment in subjects with G1, G2, and G3 voices of 100, 36, and 0% by LVS, compared to 100% using HSV via perceptual assessment. Our observations were similar; furthermore, we determined that some of the STV parameters are able to facilitate preliminary detection of cancer among organic lesions. While analyzing the STV parameters, we determined that the following can be used for this task: AmpAvg (*p* = 0.006), AmpAsymAvg (*p* < 0.001), AbsPhaseDiffAvg (*p* < 0.001), and AmpInvolvedAvg (*p* < 0.001). Their discriminative strength was acceptable as shown by the AUC ROC analysis—the highest value of 0.719 achieved by AmplAsymAvg.

Yamauchi et al. (2021) underlined that non-vibrating areas were primarily important in the detection of glottic cancers. Similar findings were described by other authors (Zhao et al. 1991; Djukic et al. 2014). Both amplitude asymmetry and decrease of average amplitude value reflect partial phonatory silence—the absence of vibration in parts of the vocal fold with the presence of malignant infiltration (Bigenzahn et al. 1998; Heyduck et al. 2022). However, the presence of a non-vibrating area, in the mentioned study, was assessed subjectively, resulting in certain problems, e.g., interrater agreement. In our study, we made an attempt to use objective parameters. Chosen parameters reflect differences in the tissue of both involved and healthy vocal folds and the effect of tissue changes on their vibrations, giving important clinical information. The amplitude of vocal fold vibrations determines the maximum distance from the center of the glottal gap that each vocal fold reaches during oscillation. In the case of infiltration of the vocal fold’s layers, it becomes stiffer, and unable to vibrate; thus, the amplitude is usually decreased in cases of deep infiltration (Svec et al. 2007; Yamauchi et al. 2021). As such, characterizing the vibration of each vocal fold with the application of quantitative analysis of HSV, using STV, parameters can provide valuable information about the texture of the tissue of the vocal fold, which can be important in the diagnosis of early glottic cancer. In our study, we determined a significant decrease in the average amplitude of the involved vocal fold in glottal cancer cases, compared to benign organic lesions (AmpInvolvedAvg, *p* < 0.001). However, other causes of decreased amplitude must also be taken into account. For example, the presence of scar tissue can result in similar non-vibrating areas as in organic tumors of the glottis (Benninger et al. 1996; Friedrich et al. 2013). That is why differentiation must be primarily based on subjective assessment of laryngeal image by experienced physician and the objective analysis can fulfill a supporting role.

Limitations of the study: research on a larger population would minimize any bias resulting from the insufficient number of participants. The study included patients with unilateral vocal fold lesions. During further studies, we plan to include patients with lesions, especially malignant, infiltrating anterior commissure with involvement of contralateral vocal fold and impairment of its function.

The study indicates that the asymmetry of the amplitude coefficient could be another parameter for detecting malignancy. It can be directly related to calculations of average amplitude as the presence of decreased vibration potential in one of the folds causes heightened asymmetry of the vocal fold oscillation amplitude. Finally, we noticed elevated phase difference between affected and non-affected vocal folds in early glottal cancers. It can be explained by the effect both mass and infiltration have on the fold—increased inertia of the involved fold, creates a delay in the phase of movement, compared to the healthy one. It can be noticed that both qualitative and quantitative analysis of HSV images deliver important data, supporting the differentiation of benign and malignant lesions of the glottis, based on functional assessment of the larynx. It can provide useful supporting tool in addition to NBI—commonly used for identification of cancerous lesions basing on their structure. Thus, disease-specific analysis seems to be a reasonable approach to the applicability of HSV in clinical practice.

## Conclusion

In conclusion, our study presents the perspectives of the application of quantified vibratory parameters (amplitude measures, glottal dynamic characteristics, and symmetry measures) derived from high-speed videolaryngoscopy as a supporting tool in the diagnosis of glottal organic lesions. We made an attempt at objectification of HSV analysis, still mostly based on subjective assessment. The comparison of phonatory movements between the healthy and involved vocal folds indicated that measurements of amplitude, asymmetry, and phase of vibrations in malignant vocal fold masses deteriorate to a significantly higher degree than in benign vocal lesions. Our results indicated that the average resultant amplitude of the involved vocal fold (AmpInvolvedAvg), average amplitude asymmetry for the whole glottis and its middle third part (AmplAsymAvg; AmplAsymAvg_2/3), and absolute average phase difference (AbsPhaseDiffAvg) can preliminarily distinguish subjects with benign from malignant vocal fold lesions. This fact reflected a perceptually observed lack or reduction in the vibratory function in the stiffer (affected by malignant lesion) vocal fold in comparison to the more pliable (healthy or affected by benign lesion) vocal fold. Thus, this method could aid preliminary differentiation between early glottic cancer and benign vocal fold masses before histopathological examination. But the golden standard remains clinical examination with videolaryngoscopy by an ENT specialist, with confirmation by histopathological examination.

## Supplementary Information

Below is the link to the electronic supplementary material.

Supplementary file1 (DOCX 14 kb)

## Data Availability

The datasets generated during and/or analyzed during the current study are available from the corresponding author on reasonable request.
