# Supplementary material for: Value of high-speed videoendoscopy as an auxiliary tool in differentiation of benign and malignant unilateral vocal lesions
Source: J Cancer Res Clin Oncol. 2024 Jan 13;150(1):10. doi: 10.1007/s00432-023-05543-y (PMC10786956; doi:10.1007/s00432-023-05543-y)
Supplement: Supplementary file 1 — Supplementary file1 (DOCX 14 kb) [file 432_2023_5543_MOESM1_ESM.docx]

| Abbreviation | Full name of the parameter | Description |
| --- | --- | --- |
| Amplitude Measures | | |
| AmpAvg (%FL) | Average glottal gap amplitude | Indicates the average resultant amplitude of vocal fold movement for the glottal gap, it’s middle third part and involved and healthy vocal fold respectively. |
| AmpAvg_2/3 (%FL) | Average amplitude of the middle third part of the glottis |  |
| AmpInvolvedAvg (%FL); AmpHealthyAvg (%FL) | Average amplitude of the involved / healthy vocal fold |  |
| Glottal dynamic characteristics | | |
| OQAvg (%); OQAvg 2/3 | Average Open Quotient for glottal gap and middle third of the glottis | Indicates the ratio of glottal opening phase to whole length of vocal cycle – average value for the whole glottal gap and middle third part respectively. Values 0-100%. 0 meaning no opening, 100% meaning no closing phase. |
| RGGA | Relative Glottal Gap Area | The ratio of minimal to maximal area of the glottis during the cycle |
| Non-opening (%FL) | Non-opening part of vocal folds | Indicates part of the glottis without opening (points along glottal axis with OQ <1%) |
| Symmetry measures | | |
| AmplAsymAvg (%); AmplAsymAvg_2/3 (%) | Average amplitude asymmetry for whole glottal gap and its middle third part respectively | This coefficient compares individual amplitudes of both vocal folds movement. 0% means that the movements are perfect reflections of each other – full symmetry; 100% means that there is no resultant vocal fold movement – they are moving in the same direction |
| PhaseAsymAvg (%);  PhaseAsymAvg_2/3  (%) | Average Phase Asymmetry for whole glottal gap and its middle third part respectively | This coefficient compares the sum of individual amplitudes of vocal fold motion to the amplitude of their resultant movement. 0% means that the resultant movement is a perfect sum of both folds movement; 100% means that there is no resultant movement. |
| AbsPhaseDiffAvg (°) | Average absolute phase difference | Absolute mean value of phase difference for whole vocal folds. |

Supplementary Table 1: Precise description of individual parameters divided into four groups: amplitude measures, glottal dynamic characteristics and symmetry measures.
